# The Role of Echocardiography in Evaluation of Takayasu’s Arteritis: A Report of Two Cases

**DOI:** 10.7759/cureus.15286

**Published:** 2021-05-28

**Authors:** Aman Patel, Subrahmanya Murti Velamakanni, Rinal M Parikh, Sapan Pandya, Tejas Patel

**Affiliations:** 1 Cardiology, Smt. Nathiba Hargovandas Lakhmichand Municipal Medical College, Ahmedabad, IND; 2 Internal Medicine, Smt. Nathiba Hargovandas Lakhmichand Municipal Medical College, Ahmedabad, IND; 3 Rheumatology, Smt. Nathiba Hargovandas Lakhmichand Municipal Medical College, Ahmedabad, IND; 4 Cardiology, Apex Heart Institute, Ahmedabad, IND

**Keywords:** takayasu's arteritis, echocardiography, thoracic aorta, abdominal aorta, subclavian artery

## Abstract

Takayasu’s arteritis (TA) is a large-vessel chronic inflammatory vasculitis that leads to thrombotic vascular occlusion. This can lead to varied presentations including limb claudication, ischemic stroke, hypertension, and heart failure. Although contrast computed tomography angiography is the main modality for imaging of the aorta and its branches, transthoracic echocardiography can be an easy-to-access, point-of-care, initial screening tool for evaluating the aorta and other cardiac structures. We present echocardiographic images from two cases that demonstrate the important cardiac structural and vascular afflictions of TA.

## Introduction

Takayasu’s arteritis (TA) is a large-vessel vasculitis mainly affecting the aorta and its major branches. It is a chronic inflammatory process that causes vascular endothelial injury and fibrosis, ultimately leading to vessel stenosis, thrombosis, dissection, and aneurysm formation [1]. Diagnosis of TA is done by the criteria described by Sharma et al., which include three major and ten minor criteria [2]. Most of the criteria for vascular involvement require contrast angiographic imaging for diagnosis. Although contrast computed tomography angiography (CTA) is the modality of choice for evaluation of the aorta and its branches, transthoracic echocardiography can be an easy-to-access important initial screening tool for evaluating the aorta and other cardiac structures. Further, heart failure due to hypertension or aortic regurgitation is one of the major causes of death in TA. Here, we present echocardiographic images from two cases that demonstrate the important cardiac structural afflictions of TA as well as imaging of the aorta using a standard Doppler echocardiography imaging probe. Echocardiography was done using the S5t and X5t imaging probes on the EPIQ-7C cardiovascular imaging system (Koninklijke Philips NV, Amsterdam, the Netherlands).

## Case presentation

Case 1

**Figure 1:**
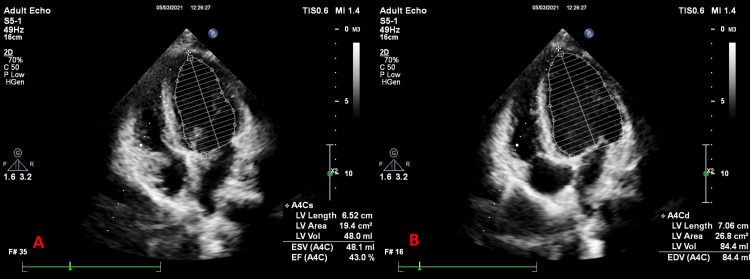
Ejection fraction calculation by Simpson’s method. (A) Apical four-chamber view in systole (A4Cs). (B) Apical four-chamber view in diastole (A4Cd). EF: ejection fraction; LV: left ventricle

**Figure 2:**
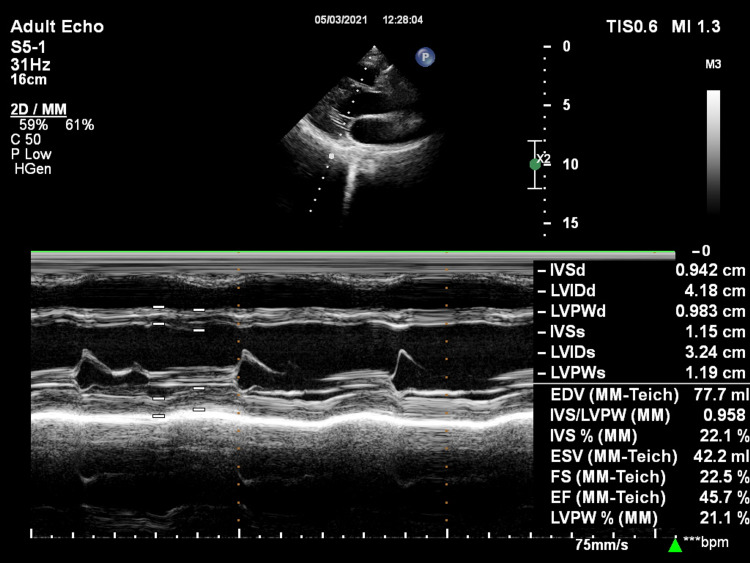
Motion-mode image in the parasternal long-axis view showing estimation of left ventricle ejection fraction. IVSd: interventricular septum diastole; LVIDd: left ventricular internal dimension diastole; LVPWd: left ventricular posterior wall diastole; IVSs: interventricular septum systole; LVIDs: left ventricular internal dimension systole; LVPWs: left ventricular posterior wall systole; FS: fractional shortening; EF: ejection fraction

**Figure 3:**
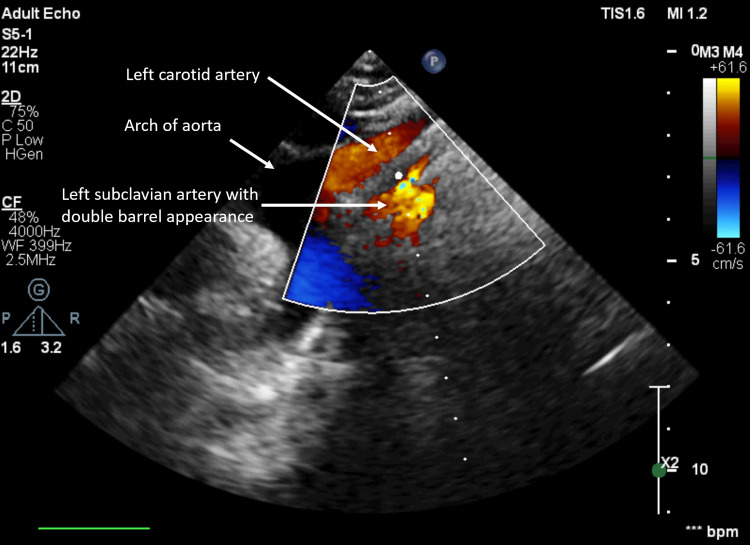
Suprasternal view with color Doppler showing the aortic arch and its main branches.

**Figure 4:**
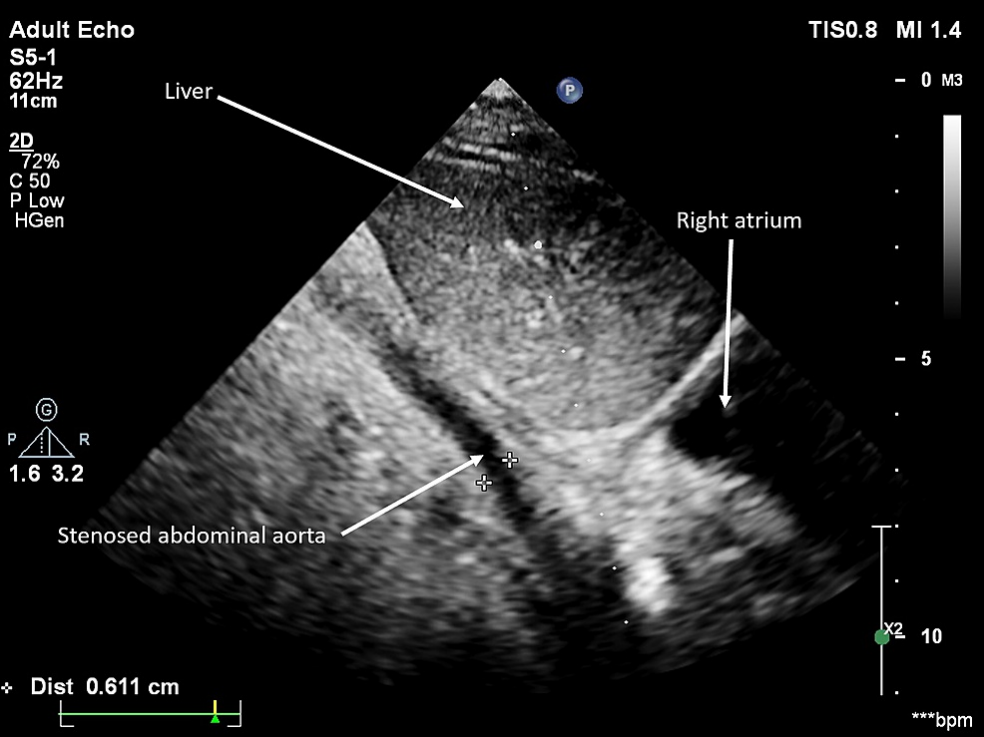
Subcostal view showing the abdominal aorta.

**Figure 5:**
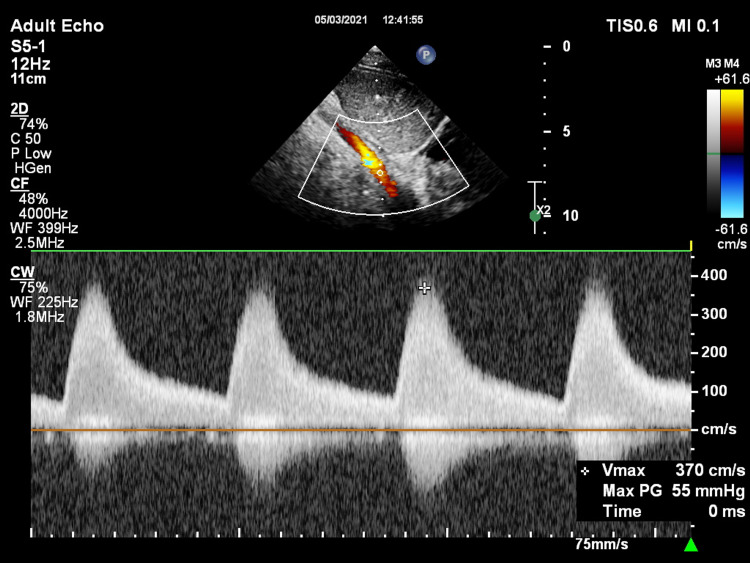
Subcostal view showing turbulent flow in the abdominal aorta with continuous wave Doppler signal showing a peak gradient of 54 mmHg suggesting stenosis. Max PG: maximum pressure gradient; Vmax: maximum velocity

A 17-year-old female presented with a history of pain in the left upper limb, headache, and New York Heart Association (NYHA) grade 2 breathlessness for 1 year. On examination, the left radial and brachial pulses were feeble. Blood pressure in the left upper limb was 150/90 mmHg and in the right upper limb was 170/100 mmHg. Cardiac auscultation was unremarkable. The patient underwent a CTA which showed left subclavian stenosis in the proximal and mid regions with dissection, as well as stenosis of the suprarenal abdominal aorta. Serum erythrocyte sedimentation rate (ESR) was 82 mm/hour (Westergren’s method). The patient had two positive major criteria of classical symptoms and left mid subclavian CTA lesion, thus satisfying the criteria for diagnosis of TA proposed by Sharma et al. [2]. On transthoracic echocardiography, the left ventricle (LV) was normal-sized with normal wall thickness. There was mild global LV hypokinesia. The LV ejection fraction (EF) was 43% by Simpson’s biplane method (Figure 1). This was correlated with the motion-mode method and visually (Figure 2, Video 1). On examination in the suprasternal window using color Doppler, turbulence and double barrel appearance were noted in the left subclavian artery just distal to its origin from the aortic arch with a peak systolic velocity of 2.2 m/s and a peak gradient of 22 mmHg (Figure 3, Video 2). On examination in the subcostal window on color Doppler, narrowing and turbulence were noted in the suprarenal abdominal aorta with a peak gradient of 54 mmHg (Figures 4, 5, Video 3). These findings were suggestive of subclavian stenosis with dissection and stenosis of the abdominal aorta.

**Video 1:** Parasternal short-axis view showing mild global left ventricular hypokinesia.

**Video 2:** Suprasternal view with color Doppler showing the aortic arch and its left main branches.

**Video 3:** Subcostal view with color Doppler showing turbulent flow in the abdominal aorta.

Case 2

**Figure 6:**
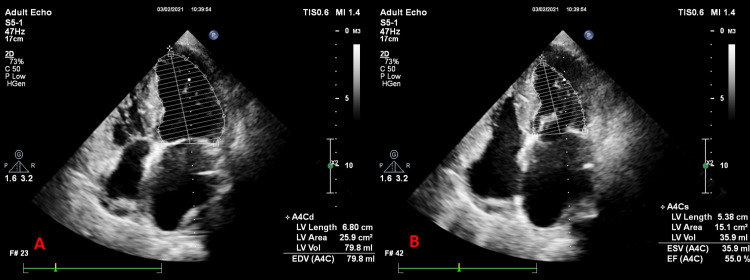
Ejection fraction calculation by Simpson’s method. (A) Apical four-chamber view in diastole (A4Cd). (B) Apical four-chamber view in systole (A4Cs). EF: ejection fraction; LV: left ventricle

**Figure 7:**
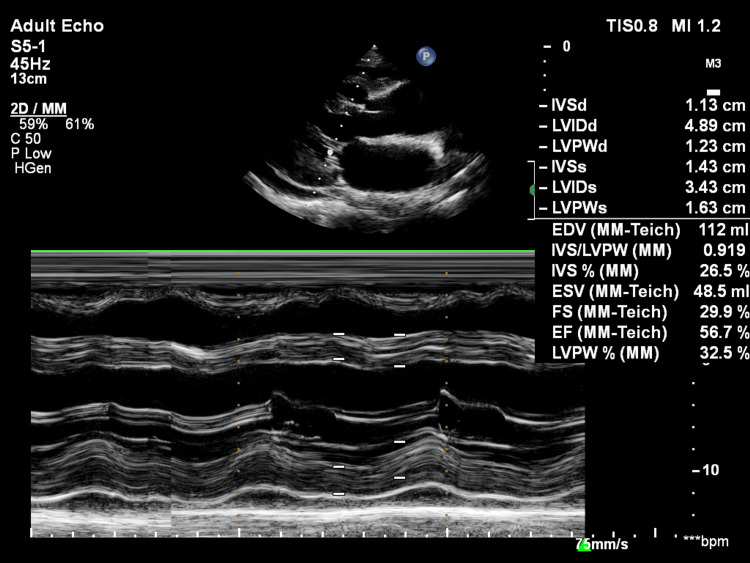
Parasternal long-axis motion mode showing left ventricular ejection fraction estimation. IVSd: interventricular septum diastole; LVIDd: left ventricular internal dimension diastole; LVPWd: left ventricular posterior wall diastole; IVSs: interventricular septum systole; LVIDs: left ventricular internal dimension systole; LVPWs: left ventricular posterior wall systole; FS: fractional shortening; EF: ejection fraction

**Figure 8:**
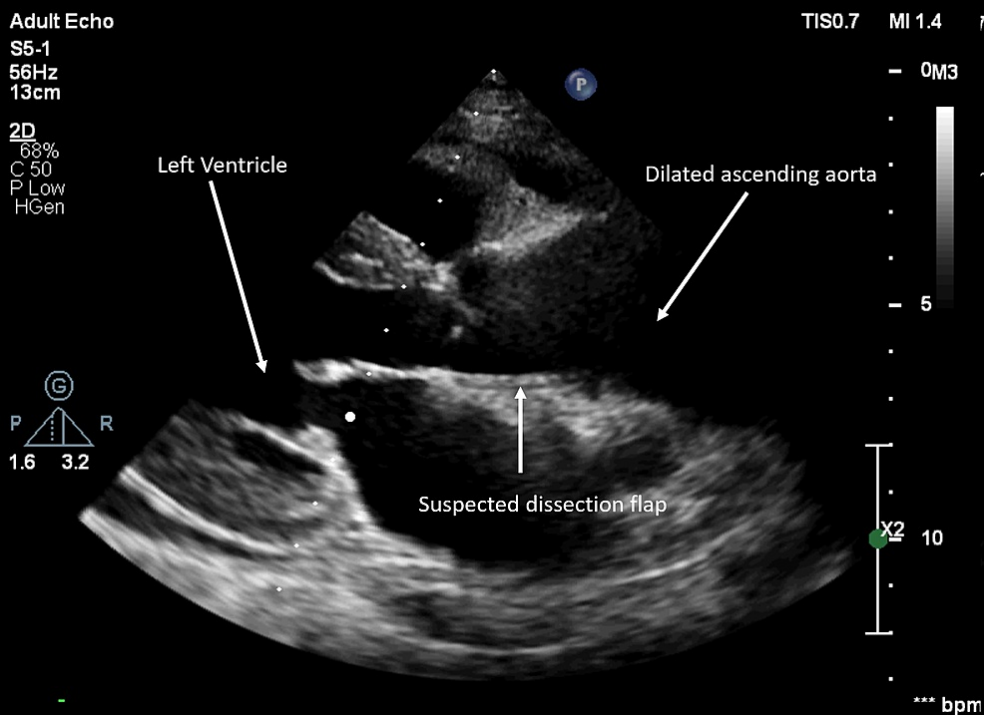
Parasternal long-axis view showing a dilated ascending aorta.

**Figure 9:**
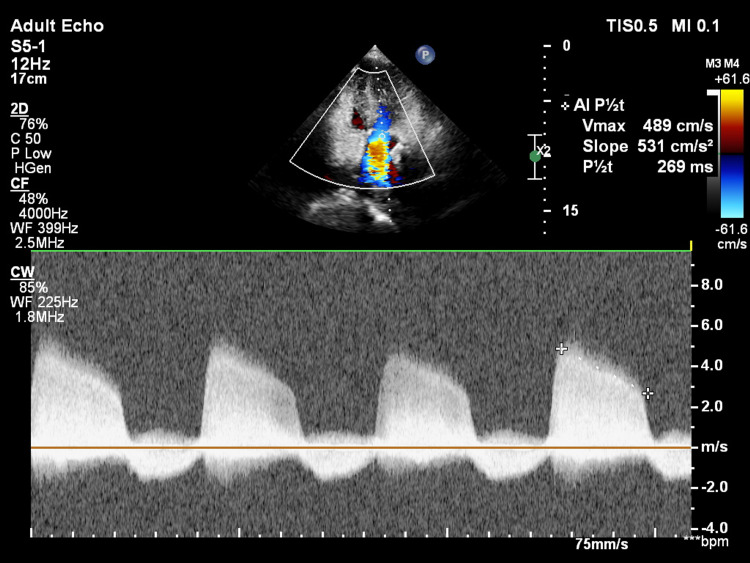
Continuous wave Doppler across the aortic valve showing an aortic regurgitation pressure half-time of 269 ms. AI P1/2t: aortic insufficiency (regurgitation) pressure half-time; Vmax: maximum velocity

**Figure 10:**
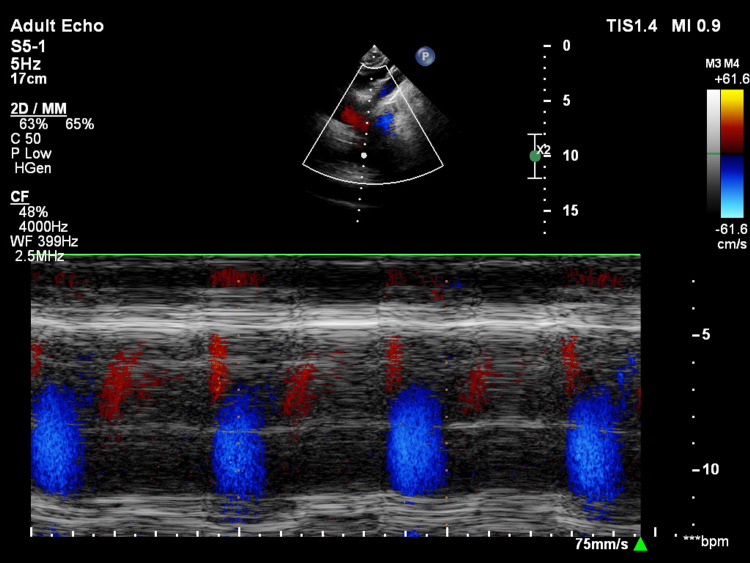
Color Doppler on motion-mode section across the descending aorta showing diastolic flow reversal.

A 38-year-old female presented with palpitations and NYHA grade 2 breathlessness for three weeks. On cardiac auscultation, a loud end-diastolic murmur with a tambour-like quality was heard in the third left interspace. Blood pressure in both upper limbs was about 180/100 mmHg. The LV apex was hyperkinetic and present in the fifth intercostal space near the mid-clavicular line. The patient underwent a CTA which showed dilation of the ascending aorta with a dissection flap. Further, there was stenosis in the suprarenal abdominal aorta. The ESR was 42 mm/hour (Westergren’s method). The patient had one positive major criterion of classical symptoms and four positive minor criteria of high ESR, aortic regurgitation, abdominal aorta lesion, and hypertension. These satisfied the conditions for the diagnosis of TA as per the criteria by Sharma et al. [2]. On transthoracic echocardiography, the LV was normal-sized, with normal EF and mild pericardial effusion (Figure 6, Video 4). Based on the values of LV cavity and wall dimensions from two dimensions and motion mode, the calculated LV mass was 220 g. Further, correcting for body surface area, the LV mass index (LVMI) was 142 g/m^2^ (reference value for females as per the American Society of Echocardiography guidelines is 43-95 g/m^2^), suggesting concentric LV hypertrophy (Figure 7) [3]. The ascending aorta was mildly dilated (40 mm maximum diameter) with suspicion of a dissection flap (Figure 8). The patient had a grade III/IV aortic regurgitation with a jet occupying around 50% of the LV outflow tract diameter (Videos 5, 6, Figure 9). There was diastolic flow reversal evident in the descending thoracic aorta (Figure 10). On examination in the subcostal window by color Doppler, turbulence was noted in the abdominal aorta suggesting stenosis (Video 7). In view of the absence of significant LV dilation, suspicious dissection flap, and a tambour-like murmur quality, consideration was given to the fact that aortic regurgitation was probably of acute onset.

**Video 4:** Parasternal short-axis view showing concentric left ventricular hypertrophy.

**Video 5:** Parasternal long-axis view showing the aortic regurgitation jet.

**Video 6:** Apical five-chamber view showing jet of aortic regurgitation.

**Video 7:** Color Doppler in subcostal view showing turbulent flow in the abdominal aorta.

## Discussion

TA was first described by the Japanese Ophthalmologist Mikito Takayasu in 1908 as a case of peculiar “wreathlike” changes in central retinal vessels in a young woman with impalpable radial pulses [4]. Since then, the disease has been extensively studied and is now recognized to be a large-vessel vasculitic thrombotic angiopathy affecting the aorta and its major branches. The global prevalence of TA has been estimated to be about 4.7-8.0 per million population with the highest in Japan being about 40 per million population [5].

The diagnostic criteria for TA have undergone many changes. Ishikawa et al. suggested the first diagnostic criteria for TA, which were modified by the American College of Rheumatology in 1990. These were then subsequently further modified by Sharma et al. in 1995 [2]. The diagnostic criteria have three major criteria and ten minor criteria. The presence of two major or one major and two minor or four minor criteria suggests a high probability of TA (Table 1). The sensitivity and specificity of the criteria by Sharma et al. are 92.5% and 95%, respectively [2]. These diagnostic criteria were further applied in an Indian study on 106 patients of proven TA and were found to be accurate [6].

**Table 1:** Diagnostic criteria for Takayasu’s arteritis by Sharma et al. [2]. ESR: erythrocyte sedimentation rate

Major criteria		
1	Left mid subclavian artery lesion	The most severe stenosis or occlusion present in the mid portion from the point 1 cm proximal to the vertebral artery orifice up to 3 cm distal to the orifice determined by angiography
2	Right mid subclavian artery lesion	The most severe stenosis or occlusion present in the mid portion from the right vertebral artery orifice to the point 3 cm distal to orifice determined by angiography
3	Characteristic signs and symptoms of at least one-month duration	These include limb claudication, absent pulses, or pulse differences in limbs, an unobtainable or significant blood presence difference (>10 mmHg systolic blood presence difference in limb), fever, neck pain, transient amaurosis, blurred vision, syncope, dyspnea, or palpitations
Minor criteria		
1	High ESR	Unexplained persistent high ESR >20 mm/hour (Westergren’s method) at diagnosis or presence of evidence in patient’s history
2	Carotid artery tenderness	Unilateral or bilateral tenderness of common arteries on palpation. Neck muscle tenderness is unacceptable
3	Hypertension	Persistent blood pressure >140/90 mmHg brachial or >160/90 mmHg popliteal
4	Aortic regurgitation or annulo-aortic ectasia	By auscultation or Doppler echocardiography or angiography
5	Pulmonary artery lesion	Lobar or segmental arterial occlusion or equivalent determined by angiography or perfusion scintigraphy, or presence of stenosis, aneurysm, luminal irregularity, or any combination in pulmonary trunk or in unilateral or bilateral pulmonary arteries determined by angiography
6	Left mid common carotid lesion	Presence of the most severe stenosis or occlusion in the mid portion of 5 cm in length from the point 2 cm distal to its orifice determined by angiography
7	Distal brachiocephalic trunk lesion	Presence of the most severe stenosis or occlusion in the distal third determined by angiography
8	Descending thoracic aorta lesion	Narrowing, dilation, or aneurysm, luminal irregularity, or any combination determined by angiography: tortuosity alone is unacceptable
9	Abdominal aorta lesion	Narrowing, dilation, or aneurysm, luminal irregularity, or aneurysm combination
10	Coronary artery lesion	Documented on angiography below the age of 30 years in the absence of risk factors such as hyperlipidemia or diabetes mellitus

Although CTA is the standard for imaging large vessels, echocardiography can also be an important adjunctive tool. The advantage of echocardiography lies in it being a point-of-care tool. It can be used bedside in patients who are sick or who have relative contraindications to contrast use such as acute kidney injury. Further, most standard echocardiography phased array probes have sufficient resolution to evaluate the proximal aorta through the suprasternal window. The abdominal aorta can also be imaged by the same probe in most cases. Hypertension is an important cause of morbidity in the TA population. Patients with TA frequently have left ventricular dysfunction and clinical heart failure. Further, aortitis can also cause significant aortic regurgitation which also leads to heart failure. Therefore, echocardiography has an important role in the workup for TA.

It is important, however, to note that echocardiography cannot replace standard CTA. Echocardiography can only image the proximal arch branches. Most lesions occur in the mid-region of the subclavian vessels which may be missed on echocardiography. Further, imaging the abdominal aorta with Doppler echocardiography probes can be difficult due to body habitus and gradients may be incorrect due to non-co-axial images.

Coronary artery involvement has been estimated to be present in about 9-11% of patients with TA with most patients having coronary ostial lesions [7]. Coronary ostia may be imaged by transthoracic echocardiography in the short-axis view at the aortic valve level. However, detailed coronary evaluation, if indicated by symptoms, needs to be done by conventional coronary angiography. A noninvasive alternative for coronary evaluation can be computed tomography coronary angiography.

There have been only a few previous studies evaluating the role of echocardiography in TA. One of the earliest studies by Tanaka et al. in 1979 was done using only motion-mode evaluation as limited by the echocardiography equipment of that time [8]. The study on 18 patients found that patients with TA may have a dilated aorta, left ventricle, and left atrium. The patients may also have thickened ventricular walls and interventricular septum, reflecting the consequences of hypertension. In another study by Turkoglu et al. from 1983 on the evaluation of left ventricular function in patients of TA, the study of 40 patients found that LV dilation and dysfunction were more common in the group with aortic regurgitation and that TA did not directly involve the heart valves or myocardium [9]. Specific signs on echocardiography like the “Macaroni sign” (diffuse homogenous vascular wall thickening) and the pseudo-coarctation sign in the aorta have also been described [10]. A recent study also evaluated the role of echocardiography as a useful noninvasive tool in evaluating the proximal pulmonary vasculature in patients with TA [11].

## Conclusions

TA has frequent cardiac involvement. Echocardiography is an important noninvasive point-of-care tool that can not only evaluate the cardiac structures but through appropriate imaging windows and planes can also help image the proximal large vessels.
